# Serum insulin-like growth factor-1 levels in females and males in different cervical vertebral maturation stages

**DOI:** 10.1590/2176-9451.20.2.068-075.oar

**Published:** 2015

**Authors:** Shreya Gupta, Anuradha Deoskar, Puneet Gupta, Sandhya Jain

**Affiliations:** 1 Senior lecturer, Index Institute of Dental Science, Department of Orthodontics and Dentofacial Orthopedics, Rau, Indore (M.P.), India; 2 Senior Lecturer, Hitkarini Dental College and Hospital, Department of Orthodontics and Dentofacial Orthopedics, Jabalpur, (M.P.), India; 3 MDS Public Health Dentistry, Government College of Dentistry, Department of Public Health Dentistry, Indore (M.P.), India; 4 Professor and Head, Government College of Dentistry, Department of Orthodontics and Dentofacial Orthopedics, Indore (M.P.), India

**Keywords:** Female, Male, Cervical vertebrae, Insulin-like growth factor 1

## Abstract

**OBJECTIVE:**

The aim of this cross sectional study was to assess serum insulin-like growth factor-1 (IGF-1) levels in female and male subjects at various cervical vertebral maturation (CVM) stages.

**MATERIAL AND METHODS:**

The study sample consisted of 60 subjects, 30 females and 30 males, in the age range of 8-23 years. For all subjects, serum IGF-1 level was estimated from blood samples by means of chemiluminescence immunoassay (CLIA). CVM was assessed on lateral cephalograms using the method described by Baccetti. Serum IGF-1 level and cervical staging data of 30 female subjects were included and taken from records of a previous study. Data were analyzed by Kruska-Wallis and Mann Whitney test. Bonferroni correction was carried out and alpha value was set at 0.003.

**RESULTS:**

Peak value of serum IGF-1 was observed in cervical stages CS3 in females and CS4 in males. Differences between males and females were observed in mean values of IGF-1 at stages CS3, 4 and 5. The highest mean IGF-1 levels in males was observed in CS4 followed by CS5 and third highest in CS3; whereas in females the highest mean IGF-1 levelswas observed in CS3 followed by CS4 and third highest in CS5. Trends of IGF-1 in relation to the cervical stages also differed between males and females. The greatest mean serum IGF-1 value for both sexes was comparable, for females (397 ng/ml) values were slightly higher than in males (394.8 ng/ml).

**CONCLUSIONS:**

Males and females showed differences in IGF-1 trends and levels at different cervical stages.

## Abstract (pt)

**OBJETIVO:**

o objetivo do presente estudo transversal foi avaliar os níveis do fator de crescimento semelhante à insulina-1 (IGF-1 sérico) em pacientes de ambos os sexos e em diferentes estágios de maturação das vértebras cervicais (MVC).

**MÉTODOS:**

a amostra consistiu de 60 pacientes, sendo 30 do sexo masculino e 30 do sexo feminino, com idades entre 8 e 23 anos. Amostras de sangue foram colhidas de todos os pacientes, cujos níveis de IGF-1 sérico foram avaliados por meio do método de imunoensaio quimioluminescente (CLIA). O estágio de MVC foi avaliado por meio de radiografias cefalométricas de perfil por meio do método descrito por Baccetti. O nível de IGF-1 sérico e o estágio de maturação das vertebras cervicais de 30 pacientes do sexo feminino foram avaliados e os dados retirados dos registros de um estudo prévio. Os dados foram submetidos aos testes de Kruskal-Wallis e de Mann-Whitney. A correção de Bonferroni foi calculada e o valor de alfa foi de 0,003.

**RESULTADOS:**

o valor de pico do IGF-1 sérico foi encontrado no estágio CS3, para mulheres, e CS4, para homens. Foram encontradas diferenças entre as médias dos valores de IGF-1 entre homens e mulheres nos estágios CS3, 4 e 5. O valor médio mais alto para os níveis de IGF-1 nos homens foi observado no estágio CS4, seguido do estágio CS5 e CS3. Nas mulheres, o valor médio mais alto foi observado em CS3, seguido do estágio CS4 e CS5. Diferenças também foram encontradas quanto à curva do IGF-1, em relação ao estágio de maturação das vértebras cervicais nos pacientes de ambos os sexos. O valor médio de IGF-1 sérico mais alto foi comparado. As pacientes do sexo feminino apresentaram valores ligeiramente mais altos (397ng/ml) em comparação aos pacientes do sexo masculino (394.8ng/ml).

**CONCLUSÕES:**

homens e mulheres apresentam valores de IGF-1 diferentes em estágios de maturação das vértebras cervicais diferentes.

## INTRODUCTION

Assessment of growth status plays a vital role in orthodontic treatment planning decisions, including cases involving the use of functional appliances, rapid maxillary expansion, retention appliances, extraoral traction forces, extraction *versus* non-extraction treatment or orthognathic surgery.^1^

Growth modification therapy carried out during the adolescent growth spurt might allow successful outcomes to be achieved within a reduced period of time.^2^ An important factor that influences timing of adolescent growth spurt is patient's sex.^3^ It is believed that the speed of adolescent spurt is lower in girls and occurs an average of 2 years earlier than boys.^4^ This aspect of growth should be taken into account while making clinical decisions. According to Hagg et al,^5^ the optimal age of maxillary expansion in girls is 12 to 13 years. However, in boys, maxillary inter-canine dimensions increase is seen until the age of 18. The clinical implication is that, in cases of crowding, any attempt to treat by expansion may not succeed due to the inability to attain a stable increase in inter-canine width. Likewise, pubertal spurt can be easily missed in early maturing girls, while in late maturers the pubertal spurt may not have started at all, but functional treatment would have been completed. Hence, determining the growth trend in each patient becomes crucial for the orthodontic practitioner.

Presently, growth assessment is carried out by means of various skeletal maturity assessing tools, such as hand wrist and cervical vertebrae radiograph.^1^ Cervical vertebrae maturation (CVM) assessed on routine lateral cephalograms protects patients from unnecessary radiation exposure by avoiding the need for additional radiograph.^6^

Nevertheless, the use of radiographic methods to predict mandibular growth has been under scrutiny for some time. It has been questioned whether mandible undergoes spurt in growth at the same time as the other skeletal structures or whether it has a late surge. In addition, CVM staging has been documented to involve subjective errors and has a decreased reproducibility.^7^ Interobserver and intraobserver disagreement exist with the same radiographs taken at different time intervals.^7^ A recent study has proposed that CVM stages cannot accurately identify the onset of the peak in mandibular growth and should be used with other methods of growth assessment.^8^ Furthermore, with incorrect neck posture while taking the radiograph, it becomes difficult to visualize the subtle changes in the vertebrae.^9^

There is evidence on accelerated mandibular growth in subjects showing radiographic skeletal maturity termed as residual mandibular growth,^10 ^and also in certain subjects before the radiographic pubertal growth stage, a phenomenon termed as juvenile acceleration.^11^ In such cases, hormonal biomarkers may provide an edge over radiographic skeletal maturity assessment methods.

Puberty is essentially a hormonal phenomenon.^12^ Biological changes that occur during puberty include several neurosecretory factors and/or hormones. The entire endocrine system is altered during adolescence, and growth hormone, thyroid and adrenal hormones are all involved in this maturational process. IGF-1 is a hormonal mediator of growth hormone.^13^ Studies have documented that serum IGF-1 level has a close association with the growth phenomenon.^13^ Hence, in order to gauge the growth status of an individual in the growth trajectory, it would be prudent to assess serum levels of biomarkers such as IGF-1.

Our previous study^14^ on serum IGF-1 on female subjects yielded encouraging results, thus, this study aimed at investigating further. The purpose of the present study was to compare the trends and levels of serum IGF-1 in female and male subjects in various CVM stages.

## MATERIAL AND METHODS

The study sample consisted of 60 subjects, 30 females and 30 males, in the age range of 8-23 years old. The study sample was randomly selected from the outpatient departments of Orthodontics and Pedodontics, Government College of Dentistry Indore, India, using the simple random sampling technique. Data of 30 female subjects (selected using the same inclusion criteria applied to the male sample) taken from records of our previous study^14^ on female subjects, carried out in the same department, were included.

All subjects were included according to the following criteria:

» Normal growth, healthy individuals (height, weight and chronological age of subjects were compared to ideal height, weight and age charts based on ICMR standards 2010 for Indian males and females.^15^ Subjects falling in the normal range were included in the study.)
» Absence of systemic disease, serious illnesses, growth abnormality, e.g. craniofacial syndromes, no bone disease or deformities, bleeding disorders or history of any serious trauma or injury to the face, hand and wrist region.
» Absence of signs of acute inflammation or infection at the time of blood sampling. No medication.
» The research protocol was approved by local Institutional Review Board. Parental/patient's informed consent form was taken prior to enrolling each subject in the study.

Lateral cephalograms were obtained and, on the same day, blood samples were collected from the median cubital vein. Time of blood sample collection for all subjects was between 12 noon and 3 pm. Serum was separated from the blood samples and labeled with a patient code (without any mention of patient's details, such as name, age and sex). It was then properly sealed and stored in a thermocol box with ice pack (kept between 2 ^o^C and 8 ^o^C ), and sent to the laboratory for chemiluminescence immunoassay for determination of IGF-1 levels by a fully automated, two-site chemiluminescent immunoassay (Siemens Immunolite 2000 immnoassay machine at Metropolis laboratories).

Lateral cephalograms were taken in natural head position. All radiographs were exposed at 80 kVp, 9 mA for 1.25 seconds. The cervical staging technique, as described by Baccetti et al,^16^ was used to stage the cervical vertebrae.

CVM staging for all samples was separately performed by two investigators at different times. Both investigators were blinded regarding patient's details, such as name, age, sex and IGF-1 levels. For all samples, the chief investigator assessed CVM stages twice at an interval of 15 days. Intraobserver reliability was 100% (Kappa = 1.0). Another senior investigator assessed the radiographs independently. Interobserver reliability was high (Kappa = 0.918). 

Data were checked for assumptions of normality by Shapiro-Wilk test. Since data did not follow normality, non-parametric tests were used. The IGF-1 levels for male and female subjects between groups were compared by means of Kruskal-Wallis ANOVA (Tables 1 and 2). Individual group differences were tested by means of Mann-Whitney test. Bonferroni correction was used for pair-wise analysis, alpha value was divided by 15 (number of comparisons) and the level of significance was set at 0.05/15 = 0.0033 (Tables 3 and 4). Data were analyzed using SPSS for 

**Table 1 -:** IGF-1 levels of female subjects at different cervical stages (n = 30).

Cervical staging	n	Mean age (years)	Mean IGF-1 (ng/ml)	SD	95% confidence interval	Median	IGF-1 Min-Max (ng/ml)
CS1	4	9.36	216 ± 7.53	8.038	208.62 - 223.38	215.5	208 - 225
CS2	6	9.8	244.33 ± 8.41	7.109	237.61 - 251.06	248.5	229 - 250
CS3	8	12.04	397 ± 20.76	9.837	382.61 - 411.39	401.5	364 - 420
CS4	5	16.14	278.8 ± 43.27	18.85	240.87 - 316.73	289	209 - 320
CS5	3	15.97	272 ± 26.15	15.58	242.40 - 301.60	260	254 - 302
CS6	4	19.49	249.25 ± 9.98	8.999	239.47 - 259.03	248	240 - 261

Kruskal-Wallis and ANOVA; significant at P < 0.000. Source: Gupta et al,14 2012.

**Table 2 -:** IGF-1 levels of male subjects at different cervical stages (n = 30).

Cervical staging	n	Mean age (years)	Mean IGF-1 (ng/ml) ± SD	Standard error	95% confidence interval	Median	IGF-1 Min-Max (ng/ml)
CS1	5	10.04	164.6 ± 36.548	15.705	132.56 - 196.64	164	107 - 200
CS2	4	11.575	214 ± 9.83	8.463	204.37 - 223.63	216	201 - 223
CS3	5	13.4	296.6 ± 55.13	23.36	248.28 - 344.92	292	236 - 369
CS4	5	14.08	394.8 ± 50.889	22.21	350.19 - 439.41	422	330 - 439
CS5	6	15.94	332.83 ± 34.45	14.84	305.26 - 360.4	339	290 - 372
CS6	5	20.28	206 ± 10.9	7.76	196.45 - 215.55	207	194 - 221

Kruskal-Wallis and ANOVA; significant at P < 0.000.

**Table 3 -:** Intergroup comparison of various cervical stages in females.

Cervical stage	Compared to stage	P value
CS1	CS2	p = 0.010 (N.S.)
	CS3	p = 0.006 (N.S.)
	CS4	p = 0.086 (N.S.)
	CS5	p = 0.034 (N.S.)
	CS6	p = 0.021 (N.S.)
CS2	CS3	p = 0.002 (Sig.)
	CS4	p = 0.100 (N.S.)
	CS5	p = 0.020 (N.S.)
	CS6	p = 0.453 (N.S.)
CS3	CS4	p = 0.003 (N.S.)
	CS5	p = 0.014 (N.S.)
	CS6	p = 0.006 (N.S.)
CS4	CS5	p = 0.456 (N.S.)
	CS6	p = 0.142 (N.S.)
CS5	CS6	p = 0.212 (N.S.)

Bonferroni's correction for 15 comparisons, alpha value set at 0.05/15=0.0033; (P < 0.0033); Sig = Significant, NS = Non significant. Source: Gupta et al,14 2012

**Table 4 -:** Intergroup comparison of various cervical stages in males

Cervical stage	Compared to stage	P value
CS1	CS2	p = 0.014 (N.S.)
	CS3	p = 0.009 (N.S.)
	CS4	p = 0.009 (N.S.)
	CS5	p = 0.006 (N.S.)
	CS6	p = 0.028 (N.S.)
CS2	CS3	p = 0.014 (N.S.)
	CS4	p = 0.014 (N.S.)
	CS5	p = 0.011 (N.S.)
	CS6	p = 0.221 (N.S.)
CS3	CS4	p = 0.047 (N.S.)
	CS5	p = 0.234 (N.S.)
	CS6	p = 0.009 (N.S.)
CS4	CS5	p = 1.000 (N.S.)
	CS6	p = 0.009 (N.S.)
CS5	CS6	p = 0.006 (N.S.)

Bonferroni's correction for 15 comparisons, alpha value set at 0.05/15=0.0033; (P < 0.0033); Sig = Significant, NS = Non significant.

## RESULTS

The IGF-1 level of subjects ranged from 107 to 439 ng/ml (total median IGF-1 = 254 ng/ml and total mean IGF-1 = 281.27 ± 80.26 ng/ml). Tables 1 and 2 give descriptive IGF-1 statistics for different cervical stages in males and females.

In males, highest mean IGF-1 value was observed in CS4 with a mean value of 394.8 ± 50.89 ng/ml at a mean age of 14.08 years. The second highest mean IGF-1 was observed at CS5 followed by CS3. The lowest mean value of IGF-1 was observed at CS1. The IGF-1 values for males with respect to cervical stages in descending order were as follows: CS4 > CS5 > CS3 > CS2 >CS6 > CS1.

In females, the highest mean IGF-1 value was observed in CS3 with a mean value of 397 ± 20.76 ng/ml at a mean age of 12.04 years. The second highest mean IGF-1 was observed at CS4 followed by CS5. The lowest mean value of IGF-1 was observed at CS1. The IGF-1 values for females with respect to cervical stages in descending order were as follows: CS3 > CS4 > CS5 > CS2 >CS6 > CS1.

Kruskal-Wallis ANOVA showed significant differences in IGF-1 levels between different cervical stages in males and female (Tables 1 and 2). Intergroup comparison was performed between different cervical stages within each sex using Mann-Whitney test with alpha at 0.0033 (with Bonferroni correction). Within females, there were statistically significant differences between CS2 and CS3, and CS3 and CS4. Among males, no statistically significant differences were found between the cervical stages at the above mentioned alpha value (Tables 3 and 4).

**Figure 1 -:**
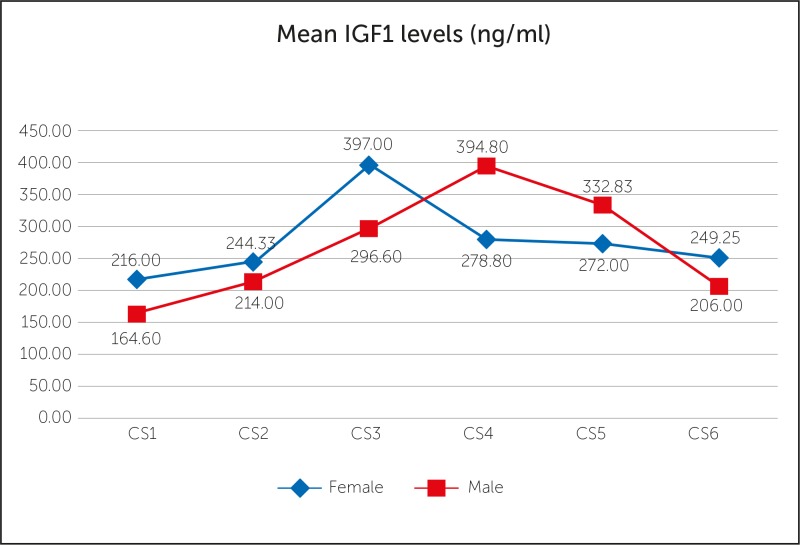
IGF-1 trends in males and females in relation to the different cervical stages.

Figure 1 demonstrates the IGF-1 trends in males and female subjects. In females, IGF-1 levels rose from CS1 towards CS2 with a spike seen from CS2 to peak at CS3, followed by a sudden decline from CS3 to CS4 continuing to CS6. In males, there was a steady increase in IGF-1 levels from CS1 to CS3 which gradually peaked at CS4, followed by slow decline to CS5 continuing to CS6.

Males and females showed differences in IGF-1 trends in relation to different cervical stages (Tables 1 and 2, Fig 1). The highest mean IGF-1 level in females was observed in CS3 followed by CS4, one cervical stage earlier than males. Chronologically, it occurred 2 years earlier in females (peak IGF-1 in females observed at a mean age of 12.04 years and at a mean age of 14.08 years in males). The greatest mean serum IGF-1 value for both males and females was compared, for females (397 ng/ml) were slightly higher than in males (394.8 ng/ml). 

## DISCUSSION

A series of investigations in the field of Medicine, Endocrinology and Dentistry have confirmed that serum IGF-1 estimation is a valid indicator of pubertal growth spurt. IGF-1 is a peptide hormone secreted primarily by the liver in response to growth hormone stimulus. During puberty, IGF-1 levels are regulated by both increased GH and sex steroids.^12^

Serum IGF-1 levels tend to peak whenever there is accelerated growth in the body whether due to the occurrence of pubertal growth spurt,^12^ adrenarche,^17^ residual mandibular growth,^10^ abnormal growth in condylar hyperplasia,^18^ acromegaly^19^ or tumorous growth occurring in the body.^20^ Furthermore, it is established that IGF-1 levels tend to be particularly sensitive with respect to growth occurring in the mandible. Studies have shown that mandibular condyle is more responsive and sensitive to IGF-1 than the femoral head.^21^

In the context of orthodontic diagnosis, treatment planning and treatment, professionals are required to know the mandible growth stage and the amount of mandibular growth that can be anticipated.

A landmark study by Baccetti et al^16^ on male and female subjects concluded that the greatest amount of mandibular growth occurred around cervical stage 3 and is the ideal stage to begin functional jaw orthopedics for correction of skeletal Class II malocclusions. It has also been documented that although boys and girls present peak growth speed at different chronological ages, cervical ages showed to be similar.^10^

However, our research data show an irregular pattern. The peak IGF-1 in male and female subjects was recorded at different chronological ages; it was also recorded at different cervical stages. IGF-1 levels increased with each subsequent cervical stages, and maximum mean values were found at CS3 in females (397 ng/ml) and at CS4 (394.8 ng/ml) in males and then decreased in later stages. These findings seem to correlate with a Turkish study^12^ in which peak IGF-1 concentrations were recorded a pubertal stage earlier in girls than boys, occurring at Tanner stage III-IV in girls and at stage IV in boys, and started to decline thereafter.

A longitudinal study by Ball et al^8^ on male subjects established a pattern of mandibular growth related to CVM stages and found that the greatest amount of mandibular growth in male subjects occurred at CS4. This is in accordance with our data which demonstrate peak IGF-1 at CS4 in male subjects.

Furthermore, our findings also correlate to some extent to the study by Ishaq et al.^22^ The study reported peak IGF-1 levels at CS4 in both males and females. Additionally, IGF-1 values in CS3 in females had higher mean values than in males, whereas CS5 in males had higher mean values than females, which corroborates our findings. In addition, the chronological age for peak IGF-1 value in male subjects in their study was found at a mean age of 14.5 years while in our study it was found at 14.08 years.

Nevertheless, in contrast, we found peak mean IGF-1 levels in female subjects at CS3 at a mean age of 12.04 years in comparison to peak value in females found at CS4 at a mean age of 14 years in the study by Ishaq et al.^22^ Discordance may be attributed to the difference in ethnic backgrounds, inclusion criteria and methodology. Also, the role of environmental and genetic factors influencing the regulation of sex steroids and IGF-1 system cannot be ruled out.

Though not statistically significant, the greatest mean IGF-1 value in females was slightly higher than males in our study. This is in agreement with the study by Brabent et al^23^ in which the authors established reference ranges of serum IGF-1 in male and female subjects separately between age groups of 1 month to 88 years. They reported slightly higher value of mean peak IGF-1 in females (410 ng/ml) as compared to males (382 ng/ml) during adolescence.

On critical assessment of IGF-1 trends, we found that in females IGF-1 levels rose from CS1 towards CS2 with a sudden rise seen from CS2 to peak at CS3, followed by a sudden decline from CS3 to CS4 continuing to CS6; while in males there was a steady increase in IGF-1 levels from CS1 to CS3, which gradually peaked at CS4 followed by a slow decline to CS5 continuing to CS6. Such findings reconfirm previous studies^4^ suggesting that females have an earlier and shorter growth spurt denoted by sharp spike and rapid decline in IGF-1 levels. Males, on the other hand, experience a later and longer growth spurt denoted by a relative plateau phase extending from CS3 to CS5 with a gradual increase and decrease in IGF-1 from CS3 to CS4 and from CS4 to CS5, respectively.

On examining the reference values of IGF-1 in both males and females in our study, the mean IGF-1 levels in cervical stages 3, 4 and 5 lie above 250 ng/ml. Considering cervical stages 3, 4 and 5 to be the stages exhibiting significant growth acceleration, as compared to the other cervical stages, it appears that mean IGF-1 level at or above 250 ng/ml indicates a period in which the individual is experiencing growth acceleration. On periodic monitoring, if IGF-1 levels accelerate or decelerate, it may suggest an upward or a downward growth trend, respectively. This hypothesis is supported by the longitudinal study by Masoud et al^24^ investigating mandibular growth and IGF-1 levels. The authors found that if IGF-1 levels have an ascending pattern above 250 ng/ml on periodic monitoring, it can be expected an average of 5.5 mm of mandibular growth, whereas if IGF-1 levels have an ascending pattern and average below 250 ng/ml, it can be expected an average of 2 mm of growth. It can be hypothesized that periodic monitoring over quarterly or 6 monthly intervals may guide the clinician regarding ascending and descending growth patterns.

To the present moment, through various studies, it has been established that biomarkers such as growth hormone (GH),^19^ IGF-1,^13^ PTHrP,^25^ sex steroids,^1^ **2 **osteocalcin,^26^ alkaline phosphatase (ALP),^26 ^etc, play an explicit role in growth phenomenon. However, out of all the suggested biomarkers, IGF-1 has shown to be the most promising marker for growth assessment.

The short half life, pulsatile secretion, diurnal variation and effects of environmental secretion stimuli make growth hormone measurements difficult.^19^ According to a recent study,^27^ serum PTHrP levels do not correlate with early pubertal stages and hence, its validity to predict peak growth is questionable. Effects of sex steroids^12^ on bone growth during adolescence are biphasic, low concentration of sex steroids in early puberty stimulate while higher concentrations inhibit bone formation. Hence, the level may lead to ambiguous assumptions.

Serum osteocalcin and alkaline phosphatase (ALP) levels correlate with pubertal stages in boys, but not in girls.^26^ Serum osteocalcin and alkaline phosphatase decrease with advancing sexual development stages (Tanner stages II - IV) in girls; however, it reaches peak levels at Tanner stage IV in boys.

It may be speculated that IGF-1 estimation could have been possible through non invasive sources such as saliva or urine. In a study by Costigan et al,^28 ^salivary IGF-1 has been shown to be extremely low, less than 1% of serum levels. In addition, gingival fluid or blood can result in inaccurate measurement. Urinary IGF-1 may demand greater patient cooperation, as it would be embarrassing for the patient and contamination of sample can also occur.

In our study, blood samples were taken for IGF-1 estimation in serum. Chemiluminescent immunoassay (CLIA) method was undertaken for IGF-1 estimation which has merits over both enzyme linked immunosorbent assay (ELISA) and radioimmunoassay (RIA). CLIA allows detection of lower analyte concentration and provides a sensitive, high throughput and economical alternative to conventional assays such as ELISA.^29^ Additionally, it does not involve hazards of preparing and handling the radioactive antigen as in RIA. CVM staging was also performed using Baccetti et al's^16^ technique which has proven to exhibit less intraevaluator and interevaluator errors when compared to other CVM staging methods.^30^ However, the drawback of our study design was that it was a cross sectional study with a limited sample size. Further studies on a large sample size is needed to establish more significant evidence even at an alpha value of 0.0033 (i.e. with Bonferroni correction).

The findings of the present study underline the fact that selection of a representative reference population is a delicate task, and that a big sample size collected from different sources reduces the risk of a non desirable impact from a single or a few subpopulations. Longitudinal studies on serum IGF-1 are needed on a larger population for deriving reference intervals, trends, amount of facial growth and the average amount of time span of the accelerated mandibular growth that occurs between males and females and different growth patterns.

## CONCLUSIONS

Males and females showed differences in IGF-1 trends and levels at different cervical stages.

IGF-1 values in CS3 in females had higher mean values than in males, whereas CS5 in males had higher mean values than females, thereby indicating earlier onset of pubertal spurt in females and more delayed and longer pubertal spurt in males.
